# Comparative Study of Hypoglycemic Effects and Mechanisms of Crude and Wine‐Processed Polysaccharides From *Polygonatum sibiricum*

**DOI:** 10.1002/fsn3.71216

**Published:** 2025-12-07

**Authors:** Hui Yang, Shuhua Zhao, Zhuoke Li, Junfeng Liu, Yang Liu, Wenbing Zhi, Hong Zhang, Tingting Sun

**Affiliations:** ^1^ Shaanxi Academy of Traditional Chinese Medicine (Shaanxi Hospital of Traditional Chinese Medicine) Xi'an People's Republic of China; ^2^ Shaanxi University of Chinese Medicine Xianyang People's Republic of China

**Keywords:** *Polygonatum sibiricum*, polysaccharide, T2DM, wine‐processing

## Abstract

Polygonatum sibiricum (PS), a traditional food and medicinal plant, primarily consists of PS polysaccharides (PSP), which have been documented to possess hypoglycemic properties. Despite the fact that wine‐processed PSP (PSPW) represents the predominant form utilized in clinical applications of PS, research has predominantly concentrated on its crude PSC (PSPC). The exact pathways responsible for the hypoglycemic effects of PSPW need further elucidation. To evaluate the hypoglycemic effects and related mechanisms of PSPC and PSPW, we applied PSPC and PSPW to an oxidative stress injury model using HepG2 cells and a T2DM rat model induced by streptozotocin (STZ). Data showed that both PSPC and PSPW can decrease the glucose oxidase (GOD) activity and reduce ROS levels in the oxidative stress injury model of HepG2 cells. Furthermore, PSPC and PSPW can not only ameliorate liver and kidney damage in T2DM rats, but also significantly improve symptoms such as FBG and weight loss. They can also reduce the activities of TG, TC, and GSP in serum, increase the activities of NEFA and INS, and upregulate the mRNA expression levels of PI3K, AKT, INSR, and GLUT‐2 in liver tissue, as well as the protein expression levels of P‐AKT, PI3K, p‐IRS1, and GLUT‐2. Moreover, the PSPW group shows a more significant improvement effect than the PSPC group. Overall, our study demonstrates that PSPC and PSPW exert their hypoglycemic effects by regulating oxidative stress and the insulin signaling pathway. Our observations aim to offer novel perspectives on PSPW's potential utilization as a therapeutic candidate and functional food components.

## Graphical abstract

PSPC and PSPW can decrease the GOD activity and reduce ROS levels in the oxidative stress injury model of HepG2 cells. PSPC and PSPW can improve T2DM by regulating oxidative and insulin signaling pathway. PSPW has better therapeutic effect on T2DM than PSPC.

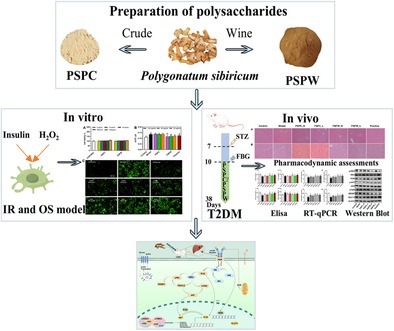

**Abbreviations**
CAT: catalase
CCK‐8: cell counting kit‐8
DM: diabetes mellitus
ECL: enhanced chemiluminescence
FBG: fasting blood glucose
GOD: glucose oxidase
GSH: glutathione
GSP: glycated serum proteins
H&E: hematoxylin and eosin
INS: insulin
MDA: malondialdehyde
NEFA: non‐esterified fatty acids
PR: Polygonatum rhizome
PS: *Polygonatum sibiricum*
PSP: *Polygonatum sibiricum* polysaccharides
PSPC: crude *Polygonatum sibiricum* polysaccharides
PSPW: wine‐processed *Polygonatum sibiricum* polysaccharides
PVDF: polyvinylidene fluoride
ROS: reactive oxygen species
SD: Sprague–Dawley
SOD: superoxide dismutase
STZ: streptozotocin
T2DM: type II diabetes mellitus
TC: total cholesterol
TG: triglyceride

## 1 Introduction

Diabetes mellitus (DM) represents a metabolic condition marked by high blood glucose (Oliveira et al. 2022) and covers type I diabetes mellitus, type II diabetes mellitus (T2DM), gestational diabetes, monogenic diabetes syndromes, disease of the exocrine, and chemical‐ or drug‐induced diabetes (ElSayed et al. 2024). Among them, T2DM is considered a global public health problem, accounting for more than 90% of all cases of diabetes. It is influenced by genetic and environmental factors, including β cell dysfunction, disturbances in glucose and lipid metabolism, chronic low‐grade inflammation, oxidative stress, ultimately resulting in insulin resistance, insufficient insulin secretion, and endocrine metabolic disorders (Li and Zhao 2023; Oliveira et al. 2022). Currently, to control blood glucose levels in patients with T2DM, a large number of synthetic anti‐diabetic medications, including sulfonylureas, biguanides, α‐glucosidase inhibitors, and glinides, have been developed and are extensively utilized to mitigate diabetic symptoms and delay the onset of complications associated with T2DM (Gong et al. 2022). Nevertheless, the prolonged use of anti‐diabetic medications can lead to various adverse effects, such as anemia, ketoacidosis, weight gain, polyuria, liver damage, renal damage, gastrointestinal discomfort, and cardiovascular complications (Kushwaha et al. 2020; Luo et al. 2019). Consequently, an urgent requirement exists for the continuous exploration of effective, low‐toxicity, natural drug alternatives (Liu, Zhang, Cai, et al. 2024). In light of this evolving scenario, optimizing diabetes treatment strategies is of paramount importance. Among the various approaches to diabetes management, the development of novel hypoglycemic agents that offer substantial glucose‐lowering effects with minimal side effects has emerged as a significant research focus (Zhang et al. 2024). Studies have demonstrated that polysaccharides derived from Chinese herbal medicine exhibit notable anti‐diabetic properties with limited adverse reactions and can effectively prevent complications (Wang et al. 2020; Yu et al. 2018). The use of naturally functional foods as dietary supplements or nutraceuticals to prevent the onset of T2DM and mitigate the complications associated with hyperglycemia has increasingly gained popularity as a cost‐effective strategy for disease management (Gong et al. 2022). Thus, identifying natural, effective anti‐diabetic agents is a promising strategy.

*Polygonatum sibiricum* (PS), commonly known as “Huangjing” in Chinese, is a traditional food and medicinal plant. The dried root of *Polygonatum sibiricum* Red., belonging to the Liliaceae family, is frequently utilized as a medicinal herb to enhance qi, nourish yin, strengthen the spleen, hydrate the lungs, and support kidney health (Chen et al. 2023; Ma et al. 2018; Sun et al. 2020). This plant is widely distributed across various regions in China, including Shaanxi, Hebei, Yunnan, Guizhou, and Sichuan, with Shaanxi noted for its most extensive distribution and highest production (Ma et al. 2018). Its initial documentation appeared in “Mingyi Biebu” during 220–245 AD, where it was recorded for its ability to enhance qi and support yin, boost spleen function, and benefit the lungs and kidneys, as well as for its use in treating diabetes, lung disease, fatigue, and feebleness (Han et al. 2020; He et al. 2022). In historical Chinese literature such as “Shen Nong Ben Cao Jing” and “Ben Cao Gang Mu”, PS is phonetically associated with “Gold”, linking it to a precious metal in vegetation, and stands recognized as a “Tonic herb” in traditional Chinese medicine. It is frequently employed to address a variety of health conditions, including weakness and respiratory ailments (Liu, Zhang, Zheng, et al. 2024). According to the modern Chinese Pharmacopeia, PS is documented to demonstrate efficacy in addressing these health issues: spleen and stomach qi deficiency, physical fatigue, stomach yin deficiency, diminished appetite, dry mouth, dry cough due to lung deficiency, and blood deficiency, thereby aiding in energy and enhancement and immune system support (Commission 2020).

Recently, multiple scientific investigations have revealed the primary constituents of Polygonatum rhizoma (PR) as polysaccharides, saponins, flavonoids, anthraquinones, and amino acids. Among these, PR polysaccharide is recognized as a key component of PS (Cui et al. 2018). It has been associated with a wide range of biological and pharmacological activities, including antioxidant, anti‐aging, anti‐fatigue, immune enhancing, antibacterial, anti‐inflammatory, lipid‐lowering, anti‐atherosclerotic, and anti‐osteoporotic, liver protective, and anti‐cancer, and hypoglycemic effects in models of diabetes induced by Alloxan or Streptozocin (Chen et al. 2023). However, the consumption of PR may lead to adverse effects such as numbness in the mouth and tongue, as well as throat irritation. Notably, these side effects are alleviated and the clinical efficacy of PR is enhanced when it is prepared with wine (Yi et al. 2017). The Chinese Pharmacopeia (2020 edition) prescribes methods for the steaming or stewing of PS with wine (Version 2020) (Commission 2020). However, the hypoglycemic properties and underlying mechanisms of polysaccharides in PSPC and PSPW are not well understood, and the rationale behind wine processing remains unclear. Additionally, the influence of PP on naturally occurring diabetes is yet to be determined.

Consequently, this study primarily sought to elucidate the molecular mechanisms by which PSPC and PSPW affect T2DM. Specifically, we assessed the effects of PSPC and PSPW on glucose uptake in insulin‐resistant (IR) HepG2 in vitro and investigated their protective effects concerning liver and kidney damage in T2DM rats. ELISA, RT‐qPCR, and Western blotting were utilized to investigate the mechanisms by which PSPC and PSPW regulate blood glucose levels in T2DM rats, both before and after wine processing of PS. The results of this study provide valuable insights into the potential application of PSPW as an innovation and establish a foundation for the use of PSP as a therapeutic agent in clinical settings, as well as a functional food ingredient with bioactive properties for the prevention and management of T2DM.

## 2 Materials and Methods

### 2.1 Materials and Reagents

Fresh *Polygonatum sibiricum* (PS) medicinal herbs were purchased from the Honghe Valley region of Meixian County, Baoji City, Shaanxi Province, China. The HepG2 human hepatic carcinoma cell line was procured from Shanghai iCell Bioscience Inc. Co. Ltd. Kits for TC, TG, NEFA, GSP, SOD, CAT, GSH, and MDA were procured from Nanjing Jiancheng Bioengineering Institute. The BCA protein assay kit was supplied by Biyuntian Biotechnology Co. Ltd. The Cell Counting Kit (CCK‐8) was supplied by Shanghai Tao Su Biochemical Technology Co. Ltd. RIPA lysis buffer and glucose oxidase (GOD) kits were provided by Beijing Solaibao Technology Co. Ltd. The DEPC‐treated and RNase‐free water was purchased from BioTeke Corporation. Trizol reagent, PrimeScript RT Kit series reverse transcription kit and TB green Premix Ex TaqTMII reagent kits were procured from Beijing Takara Co. Ltd. Primary and secondary antibodies were supplied by Zenbio Science Co. Ltd.

### 2.2 Animals

Sprague–Dawley (SD) rats, healthy males weighing 200 ± 20 g, were sourced from Chengdu Dashuo Animal Experiment Center (license number SCXK (Chuan) 2020‐030). The rats were kept in an environment with controlled temperature, humidity, and lighting, and were given a regular rodent diet and water. The Ethical Committee of Shaanxi Academy of Traditional Chinese Medicine (SYDWLL‐GL‐01) approved the study.

### 2.3 Preparation of Crude and Wine‐Processed Pieces of PS

The collected PS rhizoma (10 kg) was initially washed with water and subsequently dried at 60°C. The samples were divided into two equal portions: one designated as crude PS, and the other subjected to wine processing in accordance with the “Chinese pharmacopeia, 2015 edition (Volume IV)”, which incorporated rice wine in a volume ratio of 5:1, mixing thoroughly, and letting it expand for 12 h, followed by steaming in a pot for 4 h, until both the internal and external surfaces were uniformly moistened and turned completely black. Following this, the samples were removed, dried, and designated as wine‐processed PS (Sun et al. 2020).

### 2.4 Extraction of the PSPC and PSPW

The extraction and purification methods of PSPC and PSPW were conducted following the established protocols of our research group. Specifically, 100 g of both crude and wine‐processed PS were weighed, separately. Each sample was subjected to extraction using 12 times the amount of water, with the process involving heating and stirring at 80°C for two cycles of 2 h each time. After filtration, the combined filtrate was concentrated, and ethanol was added at 4 times the volume to facilitate precipitation over 24 h. The precipitates were then collected via centrifugation at 10,000 rpm for 10 min. Free proteins were removed using sevage reagent. After dialysis with a dialysis bag (3000 Da) and freeze‐drying, PSPC and PSPW were finally obtained (Sun et al. 2020).

### 2.5 Cell Viability Assay

Cell viability was ascertained utilizing the CCK‐8 assay with log‐phase HepG2 cells seeded into 96‐well plates at 1.0 × 10^6^ cells/mL. Following 24 h incubation at 37°C in a 5% CO_2_ incubator, the supernatants were removed. Subsequently, the cells were then exposed to increasing concentrations of PSPC and PSPW, ranging from 0 to 100 μg/mL. After an additional 24‐h incubation, cell viability was assessed using the CCK‐8 assay.

### 2.6 Cell Model Establishment and Drug Treatment

Log‐phase HepG2 cells were seeded into 96‐well plates at a concentration of 1.0 × 10^6^ cells/mL and maintained at 37°C in a 5% CO_2_ incubator with. After discarding the supernatants, 100 μL of DMEM containing 0.1 μM insulin was added to each well. The HepG2 cells were further incubated for 24 h to establish an insulin resistance model, after which drug treatments were applied as follows: (1) blank control, (2) model (0.1 μM insulin), (3) positive control (0.1 μM insulin and metformin), (4) high‐dose PSPC (0.1 μM insulin and 100 μg/mL PSPC), (5) middle‐dose PSPC (0.1 μM insulin and 40 μg/mL PSPC), (6) low‐dose PSPC (0.1 μM insulin and 20 μg/mL PSPC) (7) high‐dose PSPW (0.1 μM insulin and 100 μg/mL PSPW), (8) middle‐dose PSPW (0.1 μM insulin and 40 μg/mL PSPW), and (9) low‐dose PSPW (0.1 μM insulin and 20 μg/mL PSPW). Following a 24‐h incubation with the designated treatments, all experimental groups, with the exception of the blank control, received a supplementation of DMEM containing 10 μL of 0.3% H_2_O_2_ and were incubated for an additional 4 h. Subsequently, the cells in each group were subjected to trypsin digestion, after which RIPA cell lysate was added. The samples were then centrifuged at 10,000 rpm for 5 min at 4°C. The quantification of glucose production was performed using a glucose oxidase assay kit, while intracellular ROS levels were determined using a DCFH‐DA fluorescence assay.

### 2.7 Establishment of Rat Models of T2DM and Drug Treatment

**FIGURE 1:**
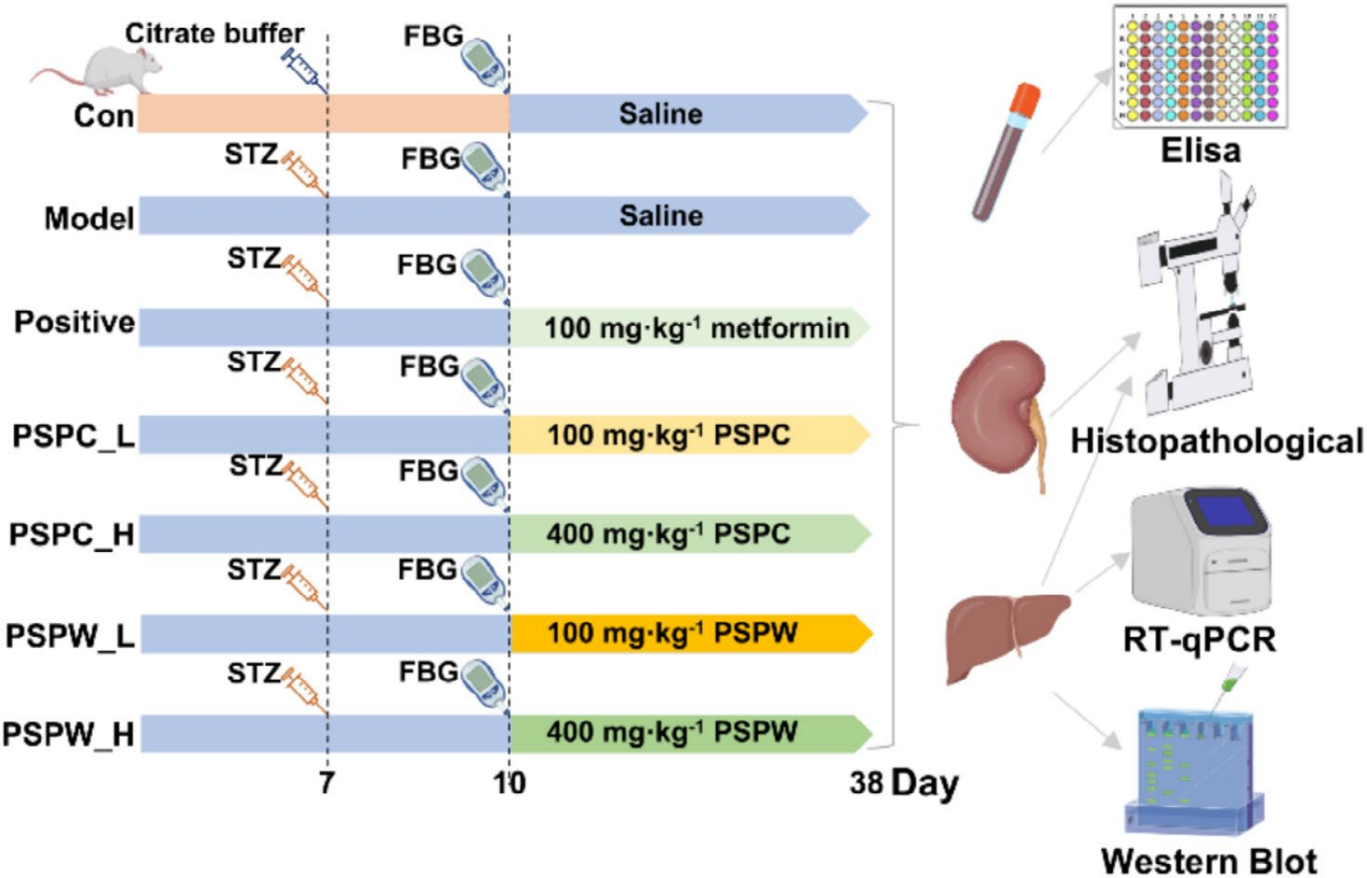
Experimental flow chart.

A total of 70 rats were randomly divided into a control group and a model group. The model group received an intraperitoneal injection of 2% streptozotocin (STZ) at a dosage of 60 mg·kg^−1^ prepared in a citrate‐buffered solution with a pH of 4.5. Following 72 h post‐injection, FBG levels were measured via tail vein sampling. An FBG level exceeding 16.7 mmol/L was used as a criterion for the successful establishment of the T2DM model. Subsequently, 60 T2DM rats were randomly assigned into six distinct groups: model group (STZ), positive group (STZ and 100 mg·kg^−1^ metformin), low‐dose PSPC (STZ and 100 mg·kg^−1^ PSPC) (PSPC_L), high‐dose PSPC (STZ and 400 mg·kg^−1^ PSPC) (PSPC_H), low‐dose PSPW (STZ and 100 mg·kg^−1^ PSPW) (PSPW_L), high‐dose PSPW (STZ and 400 mg·kg^−1^ PSPW) (PSPW_H). Each group of drugs was dissolved in saline and administered orally at a dosage of 10 mL·kg^−1^. These groups received their respective oral treatments continuously over a 4‐week period. The experimental flow chart is illustrated in Figure 1.

During the treatment period, body weight and FBG levels were recorded at five‐day intervals after subjects underwent a 12‐h fast. After the final administration, the rats underwent overnight fasting and were abdominally anesthetized with 3% pentobarbital sodium (30 mg·kg^−1^) for blood collection via the abdominal aorta. Serum was obtained by centrifuging the blood samples at 3500 rpm for 15 min. The livers and kidneys were promptly excised, rinsed, and weighed, and the liver and kidney indices were subsequently determined according to the following formulas:

$$\text{Liver index}\;\left(\%\right)=\frac{\text{Liver weight}\;\left(g\right)}{\text{body weight}\;\left(g\right)}\times 100\%$$

$$\text{Kidney index}\;\left(\%\right)=\frac{\text{Kidney weight}\;\left(g\right)}{\text{body wight}\;\left(g\right)}\times 100\%$$

Then, the serum and tissue samples were stored at −80°C for future analysis.

### 2.8 Determination of Biochemical Indicators

For further biochemical analysis, the serum samples were thawed at room temperature. The concentrations of TG, TC, FINS, GSP, and NEFA were determined using commercially available kits, following the manufacturer's instructions.

### 2.9 Histopathological Analysis

The liver and kidney tissues were preserved in a 4% formalin solution, subsequently processed through a series of ascending ethanol concentrations, cleared with xylene, and embedded in paraffin blocks. Microscopic examination was conducted on tissue sections obtained via microtome sectioning, which were subsequently visualized using hematoxylin and eosin (H&E) staining.

### 2.10 Determination of Oxidative Stress Indicators

An appropriate quantity of liver tissue was homogenized in 9 times the amount of pre‐cooled physiological saline. The homogenate was then centrifuged at 3000 rpm for 10 min, and the supernatant was collected. The levels of SOD, CAT, GSH, and MDA in the rat liver samples were measured using specific detection kits according to the manufacturer's instructions.

### 2.11 RT‐qPCR

RT‐qPCR was performed to evaluate the mRNA expression levels of PI3K, AKTA, and INSR. Total RNA was isolated using the RNA Extraction Kit (Takara) following the supplier's protocol, and subsequently converted to cDNA using the PrimeScript RT Master mix (Takara), RT‐qPCR was conducted using a real‐time PCR system. The following primer sequences were employed: GAPDH: 5′‐TGAACCCTAAGGCCAACCGTG‐3′ (forward), 5′‐ACGTACATGGCTGGGGTGTT‐3′ (reserve); PI3K: 5′‐CAGAAACTTGGGCACACAGC‐3′ (forward), 5′‐GCAGTTTTCACTTGGCACA‐3′ (reserve); AKT: 5′‐TCATTGAGGGCACCTTCCAT‐3′ (forward), 5′‐CTCCTGCCGTTTGAGTCCAT‐3′ (reserve); INSR: 5′‐GCTACCTGGCCACTATCGAC‐3′ (forward), 5′‐AACTGCCCATTGATGACGGF‐3′ (reserve). Three duplicate wells were used in each group. The relative expression levels of these genes were ascertained utilizing the relative quantitative formula 2^−ΔΔ*Ct*^.

### 2.12 Western Blotting Analysis

Liver tissues, weighing approximately 70 mg, were sectioned into small fragments and homogenized in RIPA buffer containing 1% PMSF. The homogenate was subjected to centrifugation at 12,000 rpm for 15 min at 4°C. The resulting supernatant, containing the total tissue protein, was collected, and its concentration was determined using a BCA Protein Assay Kit following the manufacturer's instructions. The protein samples were then resolved on SDS‐PAGE gels and transferred onto PVDF membranes. The membranes were blocked with 5% non‐fat milk under gentle agitation for 1 h. Primary antibodies specific to IRS1, p‐IRS1, AKT, p‐AKT, PI3K, SREBP1, and GLUT2 were applied, and the membranes were gently agitated for 1 h at room temperature, followed by overnight incubation at 4°C. Subsequently, the membranes were washed and incubated with a secondary antibody under gentle agitation for 2 h at room temperature. The relative protein expression levels were visualized using ECL and quantified with Image Lab software.

### 2.13 Statistical Analysis

The data were analyzed using SPSS 19.0 and displayed as the mean ± SD. Differences between two groups were determined using unpaired Student's *t* tests. For multiple groups comparisons, ANOVA was applied. As per convention, *p* < 0.05 and *p* < 0.01 were considered statistically significant and highly statistically significant, respectively.

## 3 Results

### 3.1 In Vitro Hypoglycemic and Antioxidant Activities of PSPC and PSPW

**FIGURE 2:**
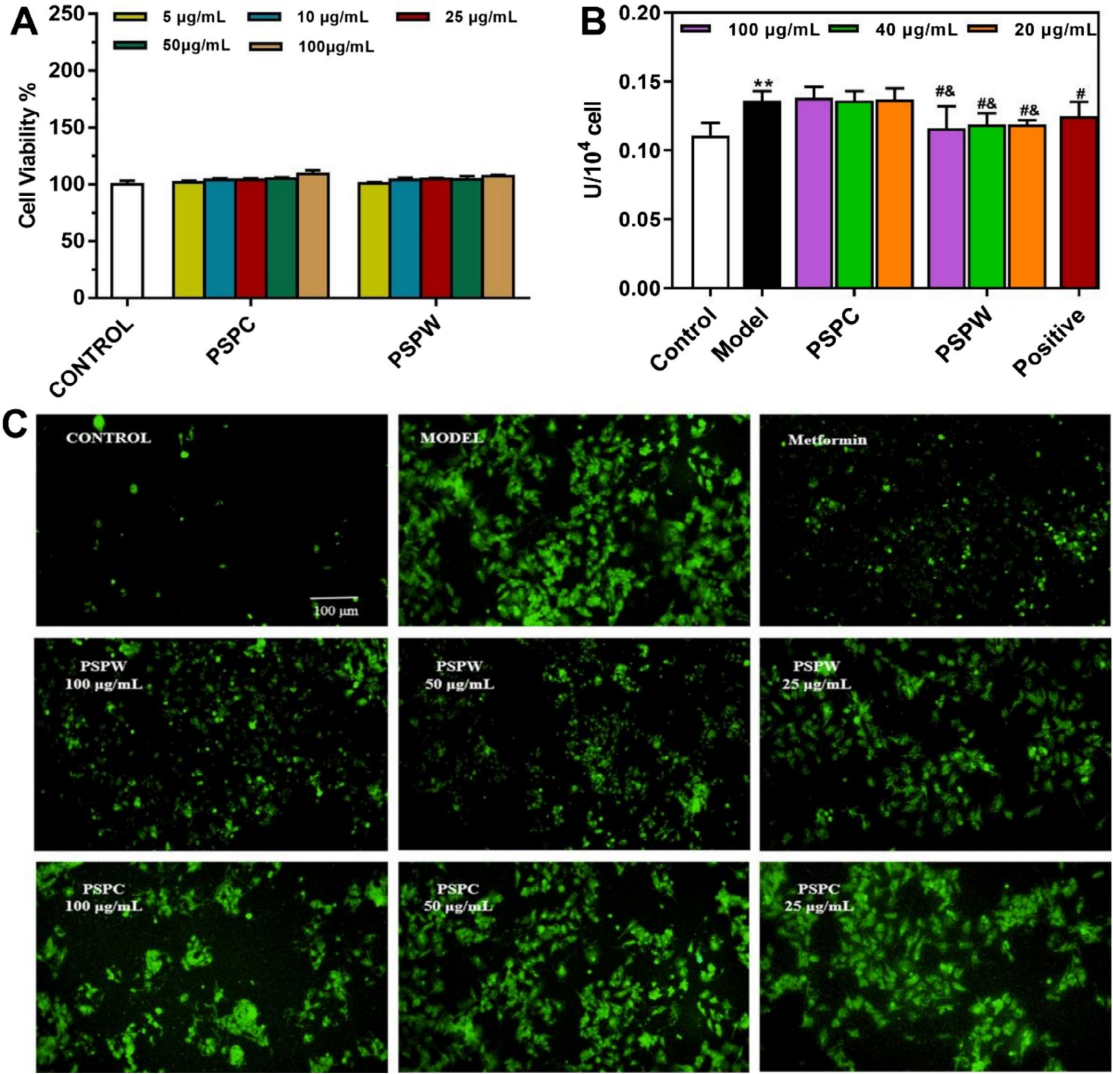
Effects of PSPC and PSPW on the insulin and H_2_O_2_‐induced IR and oxidative stress damage in HepG2 cells. (A) Cell viability (B) The content of GOD in each group. (C) ROS levels and distribution in HepG2 cells in each group under a fluorescence microscope. Data are expressed as the mean ± SD (*n* = 3). *p < 0.05 and **p < 0.01, significantly different from control; ^#^ *p* < 0.05 and ^##^ *p* < 0.01, significantly different from model; ^&^ *p* < 0.05 and ^&&^ *p* < 0.01, significantly different from PSPC.

The CCK‐8 assay evaluation of HepG2 cells treated with PSPC and PSPW at concentrations ranging from 5 to 100 μg/mL for 24 h indicated no significant impact on cell viability (Figure 2A, Table S1). In the development of a HepG2 cell model exhibiting insulin resistance (IR), a significant increase in GOD levels was observed in the model group compared to the control (*p* < 0.01) (Figure 2B, Table S2). Additionally, exposure to H_2_O_2_ resulted in a marked increase in green fluorescence within the cells of the model group relative to the control group (Figure 2C, Table S3), thereby, confirming the successful establishment of the model. Importantly, treatment of HepG2 cells with PSPC and PSPW at concentrations of 25, 50, and 100 μg/mL led to a dose‐dependent significant reduction in GOD activity in the PSPW group compared to the model group, with PSPW demonstrating a more pronounced effect than the PSPC group (*p* < 0.05) (Figure 2B, Table S2). Furthermore, following intervention with varying concentrations of PSPC and PSPW in oxidatively damaged HepG2 cells, the PSPW group exhibited a significant decrease in green fluorescence with increasing concentrations, indicating that PSPW effectively reduces ROS levels in HepG2 cells induced by H_2_O_2_ (Figure 2C, Table S3).

### 3.2 Effect of PSPC and PSPW on Body Weight, FBG, Organ Indices in T2DM Rats

**FIGURE 3:**
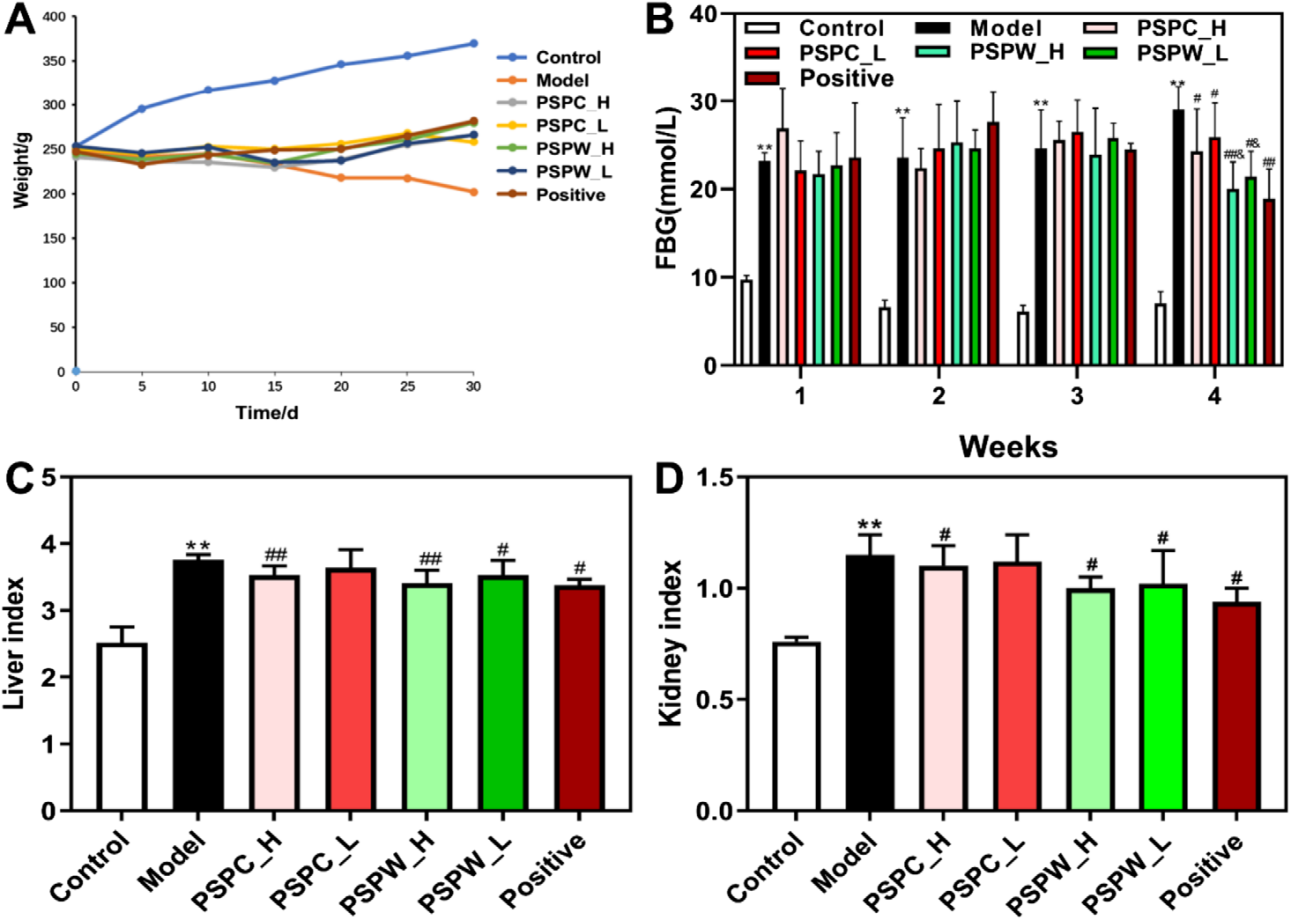
Effects of PSPC and PSPW on (A) body weight, (B) FBG, (C) liver index, and (D) kidney index in T2DM rats. Data are expressed as the mean ± SD (*n* = 9). *p < 0.05 and **p < 0.01, significantly different from control; ^#^ *p* < 0.05 and ^##^ *p* < 0.01, significantly different from model; ^&^ *p* < 0.05 and ^&&^ *p* < 0.01, significantly different from PSPC.

Following intraperitoneal administration of streptozotocin (STZ), the rats exhibited hallmark symptoms of T2DM, including polyuria, weight loss, and elevated FBG levels (Figure 3A,B, Tables S3 and S4). Additionally, there was a significant increase in the liver and kidney indices, rising from 2.52 ± 0.23 and 0.76 ± 0.02 in the control group to 3.76 ± 0.08 and 1.15 ± 0.09, respectively (*p* < 0.01) (Figure 3C,D, Table S5). Beginning on the 15th day of treatment with different concentrations of PSPC and PSPW (100 and 400 mg/kg), an upward trend in body weight was observed in the PSPW and PSPC groups compared to the model group, indicating that PSPC and PSPW may mitigate weight loss in T2DM rats (Figure 3A, Table S3). After 4 weeks of treatment, the FBG levels in the PSPC‐L, PSPC‐H, PSPW‐L, and PSPW‐H groups significantly decreased from 29.0 ± 2.7 mM to 25.9 ± 3.9 mM, 24.3 ± 4.8 mM, 21.4 ± 2.9 mM, and 20.0 ± 3.1 mM (*p* < 0.05), respectively. Notably, PSPW exhibited a more pronounced effect on reducing FBG levels compared to PSPC (Figure 3B, Table S4). Furthermore, with the exception of the PSPC‐L group, which did not significantly affect the liver index, treatment with other concentrations significantly reduced both liver and kidney indices in T2DM rats (Figure 3C,D, Table S5).

### 3.3 Effects of PSPC and PSPW on Biochemical Indicators in T2DM Rats

**FIGURE 4:**
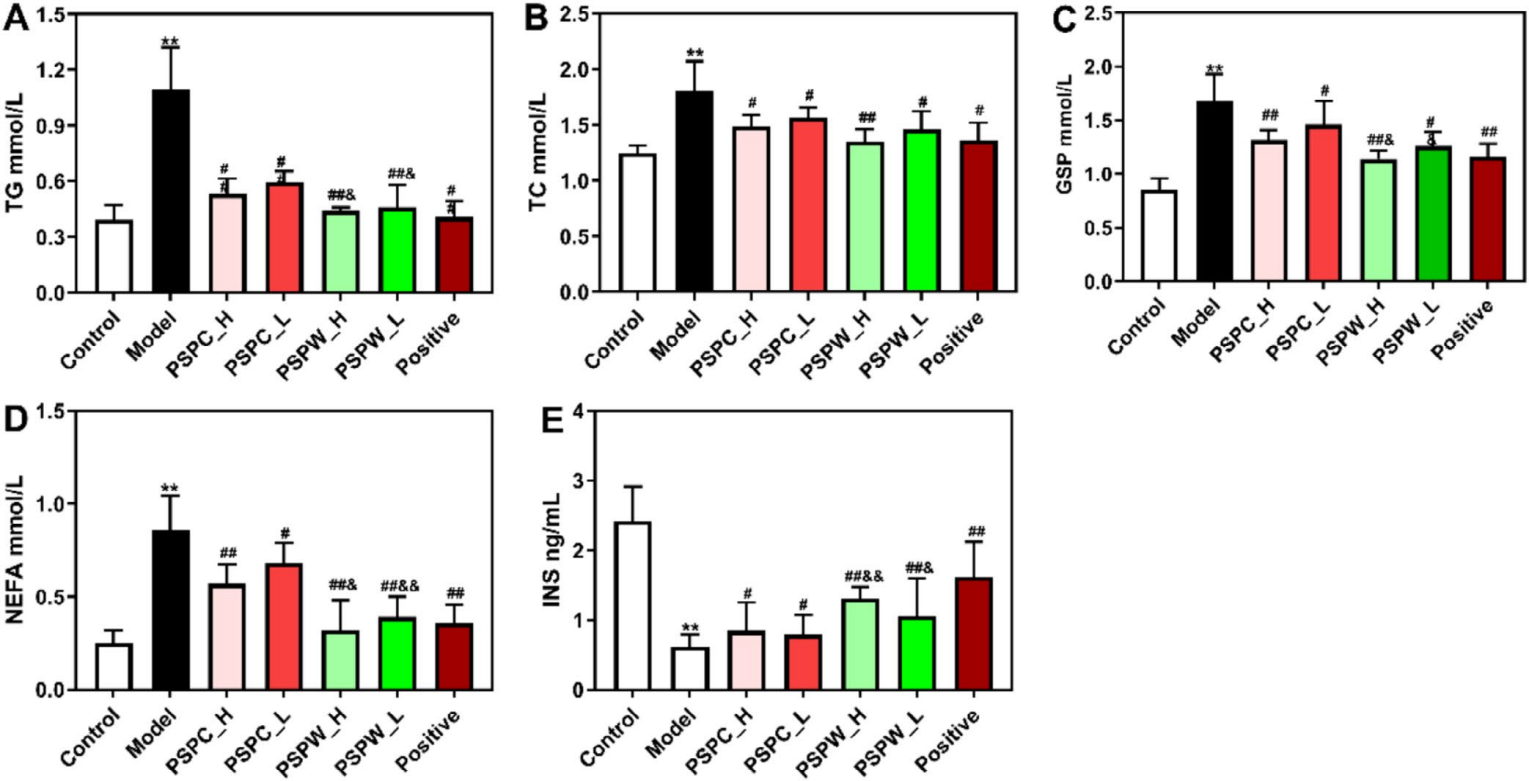
Effects of PSPC and PSPW on (A) TG, (B) TC, (C) GSP, (D) NEFA, and (E) INS in T2DM rats. Data are expressed as the mean ± SD (*n* = 9). *p < 0.05 and **p < 0.01, significantly different from control; ^#^ *p* < 0.05 and ^##^ *p* < 0.01, significantly different from model; ^&^ *p* < 0.05 and ^&&^ *p* < 0.01, significantly different from PSPC.

The effects of PSPC and PSPW on serum biochemical parameters in rats with T2DM are depicted in Figure 4. Compared to the control group, levels of TG, TC, GSP, and NEFA in T2DM rats increased significantly by 2.7, 1.1, 1.7, and 2.4 times, respectively (*p* < 0.05). Conversely, INS levels in T2DM rats decreased markedly by 74.4% compared to the control group, thereby confirming the successful establishment of the T2DM rat model. Treatment with PSPC and PSPW resulted in significant improvements in TG, TC, GSP, NEFA, and INS levels compared to untreated T2DM rats. Notably, PSPW demonstrated a more substantial effect on NEFA and INS levels than PSPC (Figure 4C,D, Table S6).

### 3.4 Effect of PSPC and PSPW on Histomorphological Alterations in Liver and Kidney

**FIGURE 5:**
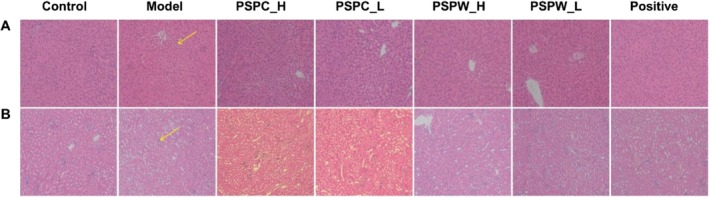
Histological changes of (A) liver and (B) kidney in different groups of rats (200× magnification).

Tissues histological analysis using H&E staining displayed that hepatocytes in the control group exhibited intact morphology and orderly arrangement (Figure 5A). Additionally, the kidney tissue displayed normal and well‐defined architecture, with clear morphology of the renal cortex and glomeruli, and no apparent abnormalities in the basement membrane or mesangial matrix (Figure 5B). Following the induction of T2DM via STZ injection, the model group exhibited hepatic steatosis, formation of lipid vacuoles, and localized hepatocyte necrosis. In kidney tissues, there was a noted increase in glomerular volume, thickening of the basement membrane, expansion of the mesangial matrix, thickening of the renal tubular basement membrane, atrophy of the majority of renal tubules, and vacuolar‐like alterations. Treatment with PSPC and PSPW significantly ameliorated these histopathological abnormalities in the liver and kidneys, indicating that both PSPC and PSPW have the potential to mitigate pathological damage in the liver and kidney tissues of T2DM rats.

### 3.5 Impact of PSPC and PSPW on Oxidative Stress Indicators in Rat Liver

**FIGURE 6:**
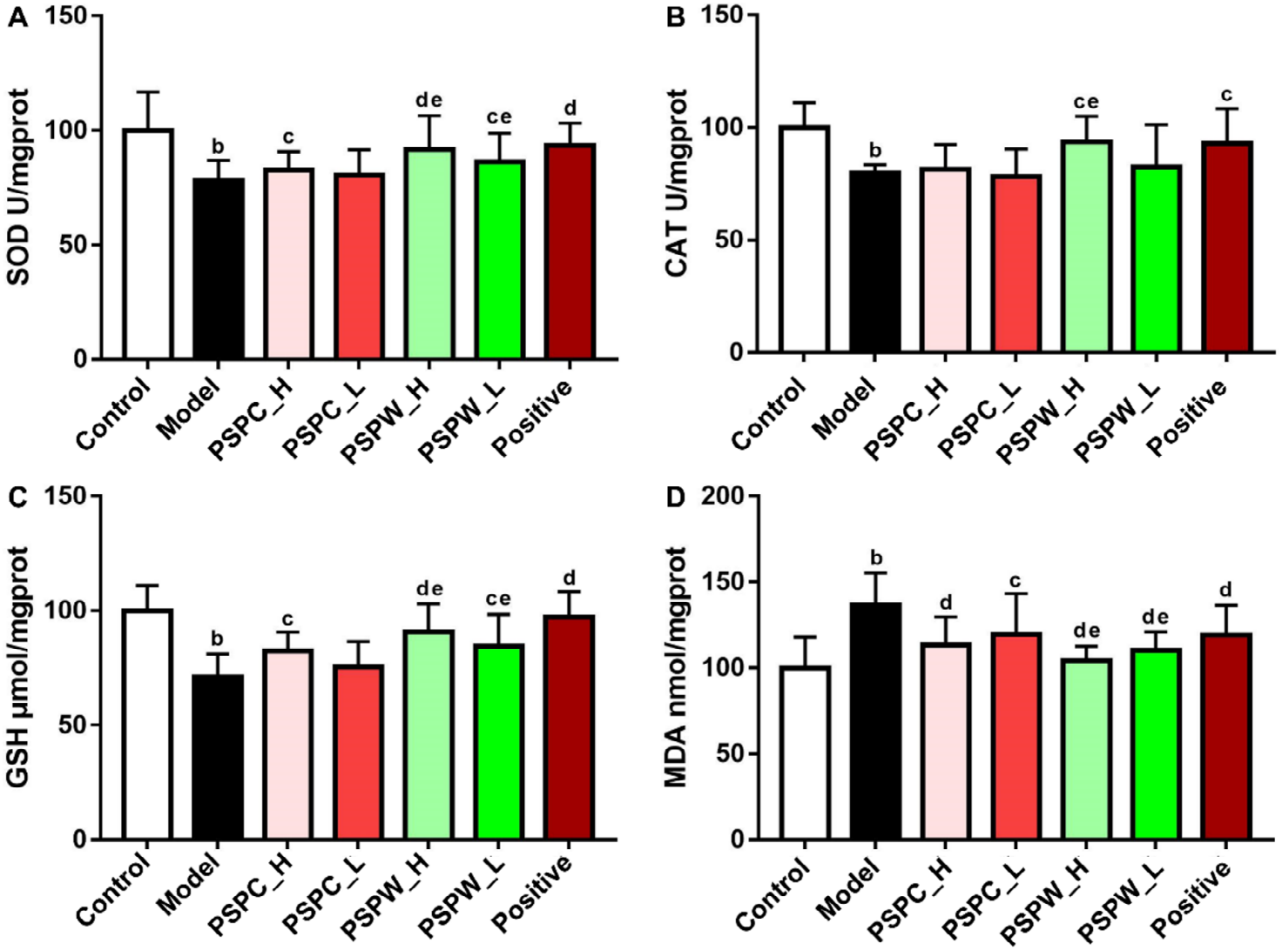
Effects of PSPC and PSPW on (A) SOD, (B) CAT, (C) GSH, and (D) MDA in T2DM rats. Data are expressed as the mean ± SD (*n* = 9). ^b^ *p* < 0.01, significantly different from control; ^c^ *p* < 0.05 and ^d^ *p* < 0.01, significantly different from model; ^e^ *p* < 0.05 and ^f^ *p* < 0.01, significantly different from PSPC.

In contrast to the control group, the liver enzymes SOD, CAT, and GSH in T2DM rats were notably reduced by 22.12%, 20.35%, and 28.92%, respectively, while MDA content was significantly elevated by 36.83% (Figure 6, Table S7). Following treatment with PSPC and PSPW, the PSPW_H groups exhibited significant reductions in SOD, CAT, and GSH enzyme activities by 15.11%, 17.53%, and 27.52% (*p* < 0.05), respectively. Additionally, MDA content in the PSPW_L and PSPW_H groups showed significant increases of 23.48% and 31.17%, respectively, in comparison to the model group. The modulating influence of PSPW demonstrated notably stronger effects in comparison with PSPC (*p* < 0.05).

### 3.6 The Expression Levels of mRNA in the Liver of Rats

**FIGURE 7:**
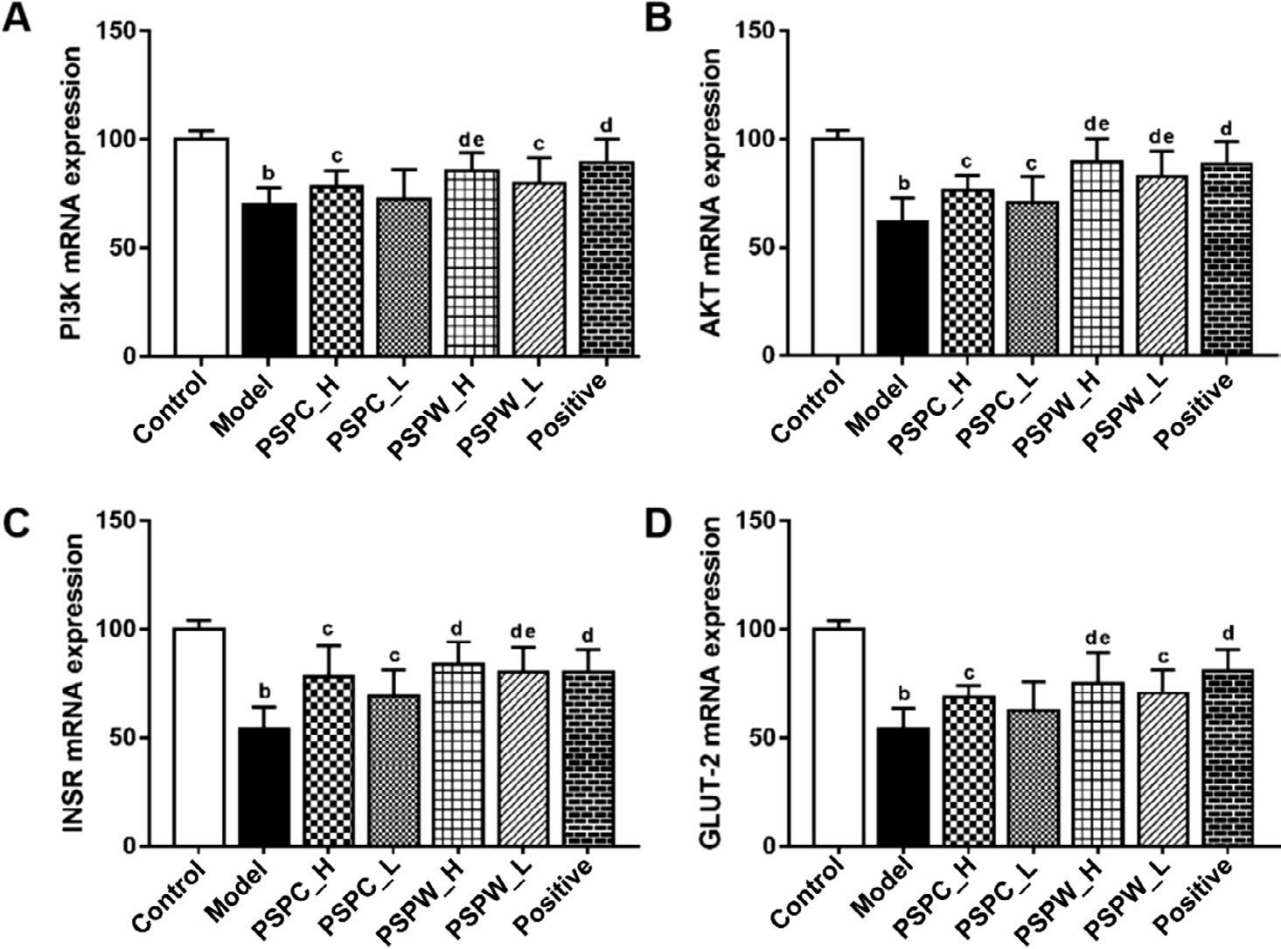
Relative mRNA expression levels of (A) PI3K, (B) AKT, (C) INSR, and (D) GLUT‐2 in rats, as determined by RT‐qPCR. All data are expressed as the mean ± SD (*n* = 3). ^b^ *p* < 0.01, significantly different from control; ^c^ *p* < 0.05, significantly different from model; ^d^ *p* < 0.05 and ^e^ *p* < 0.01, significantly different from PSPC.

To ascertain the regulatory roles of PSPC and PSPW on the expression of PI3K, AKT, INSR, and GLUT‐2 within the insulin signaling pathway, transcript levels were quantified using RT‐qPCR analysis. As shown in Figure 7 and Table S8, induction with STZ, resulted in a significant reduction in mRNA levels for PI3K, AKT, INSR, and GLUT‐2 by 1.68‐, 1.62‐, 1.84‐, and 1.85‐fold, respectively, indicating that STZ may induce T2DM through the downregulation of these mRNA expressions in rats (*p* < 0.01). Conversely, treatment with various doses of PSPC and PSPW significantly increased mRNA expression compared to the model group (*p* < 0.05), with the PSPW_H group exhibiting the most pronounced upregulation, surpassing the effects observed in the PSPC group. The evidence points to the fact that PSPW exerts a more potent ameliorative effect on T2DM, consistent with the results obtained from pharmacodynamic biochemical indicators.

### 3.7 The Expression Levels of Protein in the Liver of Rats

**FIGURE 8:**
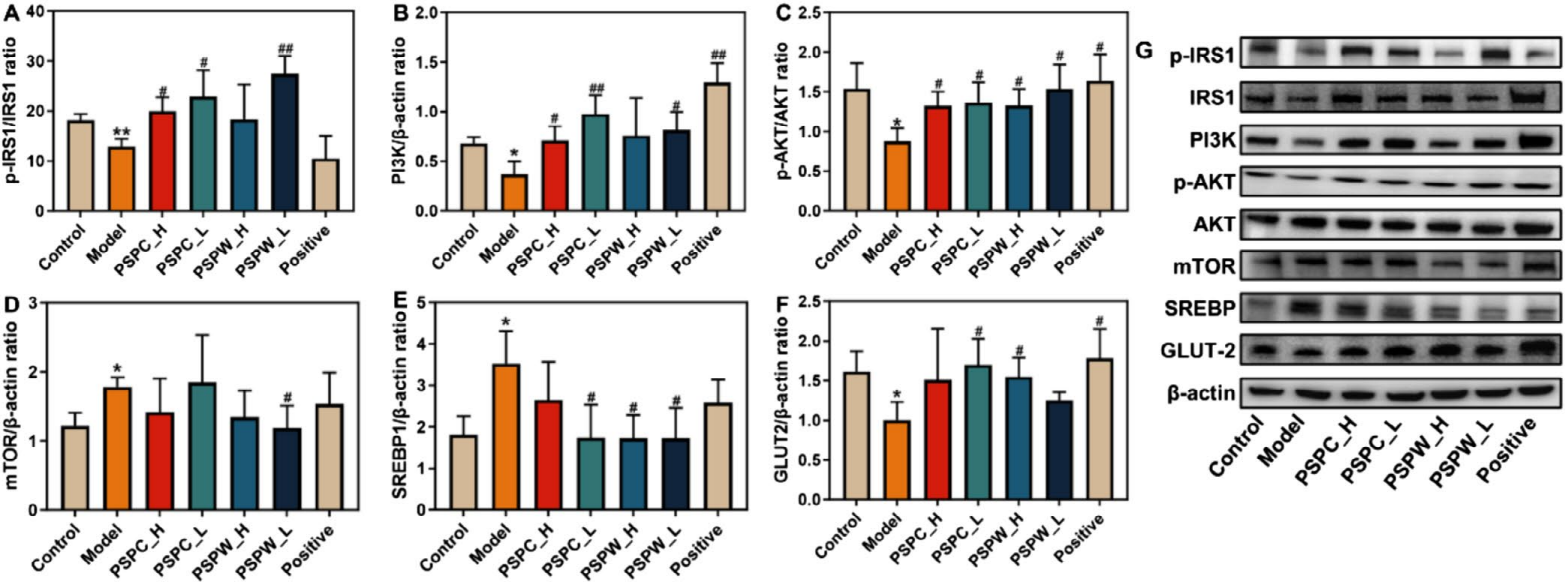
Protein expression levels of (A) p‐IRS1, (B) PI3K, (C) p‐AKT, (D) mTOR, (E) SREBP, (F) GLUT‐2, and (G) protein bands in rats. All data are expressed as the mean ± SD (*n* = 3). *p < 0.05 and **p < 0.01, significantly different from control; ^#^ *p* < 0.05 and ^##^ *p* < 0.01, significantly different from model.

To further elucidate the regulatory effects of PSPC and PSPW on the insulin signaling pathway in T2DM rats, Western blot analysis was employed to evaluate the protein expression associated with the insulin signaling pathway in the liver. The results are presented in Figure 8 and Table S9. The p‐IRS1, PI3K, p‐AKT, and GLUT‐2 levels in T2DM rats were significantly reduced compared to those in normal rats (*p* < 0.05). Conversely, the protein levels of mTOR and SREBP were significantly increased in T2DM rats relative to normal rats (*p* < 0.05). Following 4 weeks of administration of PSPC and PSPW, the p‐AKT protein levels in each treatment group were significantly higher than those in the model group (*p* < 0.05). Additionally, the levels of p‐IRS1 and PI3K in the PSPC_L, PSPC_H, and PSPW_L groups were markedly elevated relative to the model group (*p* < 0.05). The expression levels of GLUT‐2 protein in the PSPC_L and PSPW_H groups were notably higher than those in the model group (*p* < 0.05). In contrast, the expression levels of SREBP protein in the PSPC_L, PSPW_H, and PSPW_L groups were significantly reduced compared to the model group (*p* < 0.05), with a reduction in mTOR protein expression observed only in the PSPW_L group compared to the model group (*p* < 0.05). However, no major differences were found between the PSPW and PSPC treatments.

## 4 Discussion

To date, although the hypoglycemic effects of PSP have been well documented, there is insufficient research regarding its hypoglycemic effects and mechanisms following wine processing in clinical practice. According to the concept of processing of Traditional Chinese Medicine (TCM), various processing techniques, such as steaming using alcohol or heating with vinegar, are commonly employed to mitigate toxicity and enhance therapeutic effectiveness (Peng, Hu, et al. 2022).

Studies have demonstrated that the oligosaccharide composition of PS undergoes significant modification after wine roasting, resulting in enhanced antioxidant activity compared to pre‐wine‐roasted PS, as demonstrated in both in vitro and in vivo (Wang et al. 2024). Furthermore, oxidative stress has been implicated in a wide range of human diseases, with STZ‐induced diabetes being a consequence of oxidative stress (Adefegha et al. 2022; Chen, Wang, et al. 2024). Given the role of polysaccharides in alleviating oxidative stress through their antioxidant properties (Adefegha et al. 2022; Chen, Wang, et al. 2024), we conducted an in vitro antioxidant study and assessed oxidative stress‐related indicators, including MDA, SOD, CAT, and GSH. In our study, we observed that both PSPC and PSPW exhibit antioxidant activity, with PSPW demonstrating superior efficacy compared to PSPC. This finding aligns with the investigation conducted by Peng et al. (2021), which suggests that wine roasting enhances the antioxidant capacity of polysaccharides. Furthermore, our previous studies have consistently shown that PSPW exhibits greater antioxidant activity than PSPC, particularly in terms of free radical scavenging (Sun et al. 2023). There are studies showing that the antioxidant activity of polysaccharides is associated with their higher molecular weight and uronic acid content. Our previous study found that the molecular weight of PSPW (1.42 × 10^4^ Da) is higher than that of PSPC (4.01 × 10^3^ Da). The galacturonic acid content of PSPW (13.84%), exceeds that of PSPC (10.20%) (Sun et al. 2020). Similarly, Su et al. demonstrated that the antioxidant capacity of four types of Auricularia polysaccharides increased with higher molecular weight (Su and Li 2020). Peng, Song, et al. (2022) also reported that polysaccharides with elevated molecular weight and uronic acid content in Camellia fascicularis leaves exhibit enhanced antioxidant activity. In addition, we found that both PSPC and PSPW significantly decreased the levels of TC and TG in T2DM in this study, because IR is marked by a reduced capacity of target tissues to take up fatty acids. Consequently, a substantial amount of free fatty acids is released into the blood and transported to the liver, providing components for TG and TC (Wang et al. 2023). In a related study examining the hypoglycemic effects of various processed products of PS, Zhang (2019) observed that wine‐processed PS also effectively reduced TC and TG levels in hyperglycemic mice.

An increased FBG level is another critical symptom of T2DM (Ti et al. 2022). In this study, we observed that the PSPC, PSPW and the positive group exhibited minimal effects on FBG during the first 3 weeks, with a significant decrease observed until the fourth week. This may be due to the fact that T2DM is a chronic disease; the FBG in the model group continued to rise, and insulin resistance had not been fully alleviated in the early stages of treatment. Consequently, no significant difference in FBG was observed between the drug treatment group and the model group during the initial 3 weeks. In their investigation of berberine's therapeutic mechanism for T2DM, Di et al. observed a progressive increase in FBG levels within the model group. Conversely, the medication group exhibited a significant increase in FBG during the second week, followed by a notable decrease by the fourth week (Di et al. 2021), findings that align with the results of our study.

**FIGURE 9:**
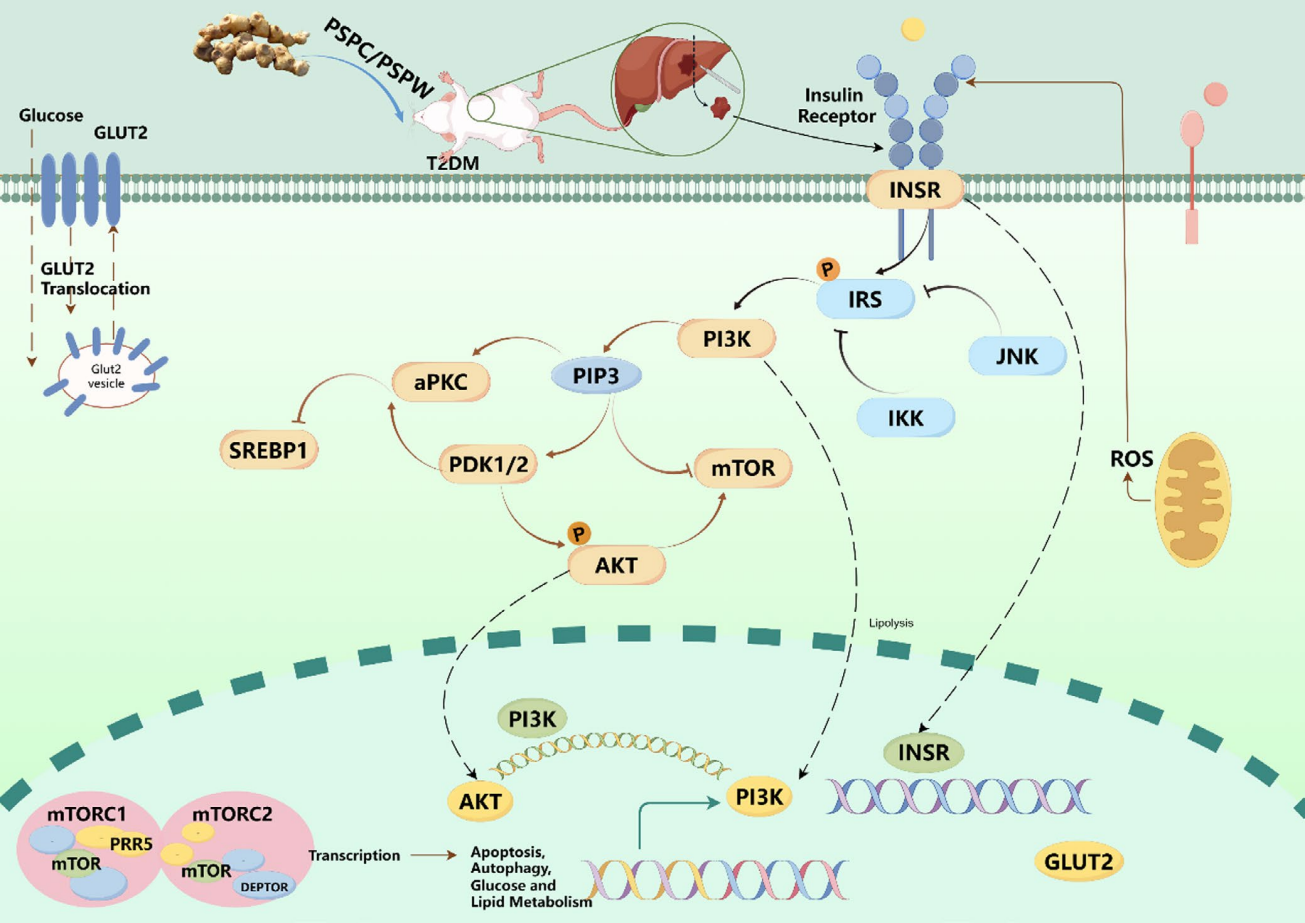
Possible hypoglycemic mechanisms of PSPC and PSPW on T2DM rats (by Figdraw).

Insulin is pivotal in maintaining energy homeostasis among the various hormones that regulate metabolic pathways. Disruption in insulin signaling can lead to an imbalance in energy homeostasis, resulting in various health complications (Li et al. 2021). This study provides preliminary evidence that PSPW exerts a hypoglycemic effect through the modulation of oxidative stress and the insulin signaling pathway. Upon oral administration of PSPW to T2DM rats, there was an initial activation of insulin receptors within the insulin signaling pathway, leading to the upregulation of p‐IRS1 and subsequently increasing the expression levels of PI3K. This upregulation further enhanced the expression of AKT while reducing the expression of mTOR. Additionally, the expression of GLUT2 was increased, and the expression of SREBP was decreased, collectively contributing to the amelioration of T2DM rats (Figure 9). The modulation of PI3K may be attributed to the synergistic effects of insulin and receptor activation. Subsequently, PI3K stimulated downstream of the insulin receptor substrate (IRS) can enhance AKT phosphorylation. By regulating glucose levels in the liver, the cell membrane plays an essential role in reducing insulin resistance and ameliorating the symptoms of T2DM. AKT facilitates the translocation of glucose transporter vesicles to the cell membranes, thereby maintaining blood glucose homeostasis (Wang et al. 2023). Furthermore, the activation of the PI3K/AKT pathway can also stimulate glycogen synthesis (Huang et al. 2022). As a critical signaling protein downstream of PI3K, AKT regulates lipid and glucose metabolism, and is prominently expressed in insulin‐responsive tissues such as the liver, muscle, and adipose tissue (Chen, Jiang, et al. 2024). The current study highlights the role of PSPC and PSPW in enhancing the expression of PI3K and p‐AKT in T2DM rats. These results demonstrate that PSPC and PSPW can promote intracellular glucose transport and stimulate glycogen synthesis. Recent studies have indicated that the dysregulation of mTOR is associated with T2DM (Amin et al. 2024). Several studies have indicated that the overactivation of the mTOR pathway can disrupt the IRS1/PI3K/AKT signaling pathway, resulting in hepatic gluconeogenesis and glycogen breakdown, while also inhibiting glucose uptake in muscle and adipose tissue, thereby exacerbating T2DM and IR (Bu et al. 2025). These findings are consistent with the results of the current study, which demonstrate that PSPC and PSPW can effectively suppress mTOR protein expression in T2DM rats, thereby ameliorating IR and contributing to the treatment of T2DM. PSPC and PSPW enhance glucose uptake and utilization, as well as improve glucose and lipid metabolism, by significantly increasing the expression of GLUT2 in T2DM rats. This effect may be attributed to the ability of polysaccharides to enhance cellular glucose uptake and utilization by promoting the membrane translocation or upregulation of GLUT2 expression. Notably, when GLUT2 expression was knocked down, the regulatory effects of PSPC and PSPW on glucose and lipid metabolism, as well as OS disorders in diabetes, were significantly diminished (Zhang and Ning 2025).

Although this study demonstrates that both PSPW and PSPC are effective in treating T2DM, and PSPW exhibits greater activity than PSPC. The structural characteristics of polysaccharides fundamentally underpin their biological activity. Consequently, the biological effects of polysaccharides are influenced not only by their physicochemical properties, relative molecular weight, and sugar composition, but also by their functional groups, degree of branching, length of the main and branch chains, three‐dimensional structure, and mode of sugar linkage (Tan et al. 2017). Despite this understanding, the specific structural attributes of polysaccharides that confer hypoglycemic effects in PSPW remain inadequately elucidated. Furthermore, it is uncertain whether the wine processing of PS induces reactions that generate new substances capable of enhancing hypoglycemic activity. Therefore, further research should focus on elucidating the structure of polysaccharides in PSP, their structure–activity relationship concerning hypoglycemic effects, and the interplay between processing mechanisms and biological activities.

## 5 Conclusion

In this present study, PSP has been shown to exert antioxidant and hypoglycemic effects by reducing GOD and ROS concentrations in HepG2 cell models with IR and OS damage. Furthermore, by improving the organ index and pathological damage of the liver and kidneys, as well as TG, TC, INS, GSP, and FBG levels in serum, SOD, CAT, GSH enzyme activities, and MDA content in the liver of T2DM rats, PSP exhibited its therapeutic effect on T2DM rats. Nevertheless, the PSPW demonstrated better hypoglycemic activity than the PSPC. Using RT‐qPCR and Western blot analysis, we established that PSP improves T2DM conditions by modulating the mRNA and protein expression levels of IRS1, PI3K, AKT, SREBP1, and GLUT2 in the insulin signaling pathway. Overall, this investigation not only establishes a foundation for examining the hypoglycemic properties of PSP, but also confirms the therapeutic potential of PSPW and provides data support for its clinical application. Moreover, considering PS's dual role as both a medicinal and food source, these findings contribute valuable insights for the development of functional food products incorporating PSP for the management and prevention of T2DM.

## Author Contributions

Tingting Sun: funding acquisition, review and editing. Hong Zhang: conceptualization, methodology. Hui Yang and Shuhua Zhao: data curation, formal analysis, software, writing – original draft. Zhuoke Li and Junfeng Liu: visualization, investigation. Yang Liu and Wenbing Zhi: supervision. The final manuscript was reviewed and approved by all authors.

## Conflicts of Interest

The authors declare no conflicts of interest.

## Supporting information


**Table S1:** The effects of PSPC and PSPW on the viability of HepG2 cells. Data are expressed as mean ± SD (*n* = 3).
**Table S2:** The effects of PSPC and PSPW on GOD levels in HepG2 cells with insulin resistance and oxidative damage. Data are expressed as mean ± SD (*n* = 3).
**Table S3:** Effects of PSPC and PSPW on body weight in T2DM rats. Data are expressed as mean ± SD (*n* = 9).
**Table S4:** Effects of PSPC and PSPW on FBG in T2DM rats. Data are expressed as mean ± SD (*n* = 9).
**Table S5:** Effects of PSPC and PSPW liver and kidney index in T2DM rats. Data are expressed as mean ± SD (*n* = 9).
**Table S6:** Effects of PSPC and PSPW on TG, TC, GSP, NEFA, and INS in T2DM rats. Data are expressed as mean ± SD (*n* = 9).
**Table S7:** Effects of PSPC and PSPW on SOD, CAT, GSH, and MDA in T2DM rats. Data are expressed as mean ± SD (*n* = 9).
**Table S8:** Relative mRNA expression levels of PI3K, AKT, INSR, and GLUT‐2 in rats, as determined by RT‐qPCR. Data are expressed as mean ± SD (*n* = 3).
**Table S9:** Protein expression levels of p‐IRS1, PI3K, p‐AKT, mTOR, SREBP, and GLUT‐2 in rats. Data are expressed as mean ± SD (*n* = 3).

## Data Availability

The datasets generated for the current study are available from the corresponding author upon reasonable request.
