# Supplementary material for: Comparative Study of Hypoglycemic Effects and Mechanisms of Crude and Wine‐Processed Polysaccharides From Polygonatum sibiricum
Source: Food Sci Nutr. 2025 Dec 7;13(12):e71216. doi: 10.1002/fsn3.71216 (PMC12682707; doi:10.1002/fsn3.71216)
Supplement: Supplementary file 1 — Table S1: The effects of PSPC and PSPW on the viability of HepG2 cells. Data are expressed as mean ± SD (n = 3). Table S2: The effects of PSPC and PSPW on GOD levels in HepG2 cells with insulin resistance and oxidative damage. Data are expressed as mean ± SD (n = 3). Table S3: Effects of PSPC and PSPW on body weight in T2DM rats. Data are expressed as mean ± SD (n = 9). Table S4: Effects of PSPC and PSPW on FBG in T2DM rats. Data are expressed as mean ± SD (n = 9). Table S5: Effects of PSPC and PSPW liver and kidney index in T2DM rats. Data are expressed as mean ± SD (n = 9). Table S6: Effects of PSPC and PSPW on TG, TC, GSP, NEFA, and INS in T2DM rats. Data are expressed as mean ± SD (n = 9). Table S7: Effects of PSPC and PSPW on SOD, CAT, GSH, and MDA in T2DM rats. Data are expressed as mean ± SD (n = 9). Table S8: Relative mRNA expression levels of PI3K, AKT, INSR, and GLUT‐2 in rats, as determined by RT‐qPCR. Data are expressed as mean ± SD (n = 3). Table S9: Protein expression levels of p‐IRS1, PI3K, p‐AKT, mTOR, SREBP, and GLUT‐2 in rats. Data are expressed as mean ± SD (n = 3). [file FSN3-13-e71216-s001.docx]

**Supplementary information**

**Comparative Study of Hypoglycemic Effects and Mechanisms of Crude and Wine-Processed Polysaccharides from *Polygonatum sibiricum***

Hui yang^a1^, Shuhua Zhao^b1^, Zhuoke Li^a^, Junfeng Liu^a^, Yang Liu^a^, Wenbing Zhi^a^, Hong Zhang^a*^, Tingting Sun^a*^

^a^ Shaanxi Academy of Traditional Chinese Medicine (Shaanxi hospital of traditional Chinese medicine), Xi’an 710003, PR China

^b^ Shaanxi University of Chinese Medicine, Xianyang, 712046, PR China

^1^ These authors contributed equally to this work.

* Corresponding authors: Hong Zhang E-mail: zhanghong919919@163.com

Tel: 029-85395696

Tingting Sun E-mail: sttlt@126.com

Tel: 029-85395696

**Table** **S1** The effects of PSPC and PSPW on the viability of HepG2 cells. Data are expressed as mean ± SD (n = 3).

| Group | Concentration (µg/mL) | Cell viability (%) |
| --- | --- | --- |
| Control | - | 100 |
| PSPW | 5 | 104.84 ± 0.52 |
|  | 10 | 105.33 ± 1.35 |
|  | 25 | 107.96 ± 2.88 |
|  | 50 | 107.59 ± 4.45 |
|  | 100 | 107.91 ± 4.55 |
| PSPC | 5 | 104.67 ± 3.65 |
|  | 10 | 104.92 ± 5.23 |
|  | 25 | 101.75 ± 2.75 |
|  | 50 | 106.88 ± 3.56 |
|  | 100 | 107.24 ±3.03 |

**Table** **S2** The effects of PSPC and PSPW on GOD levels in HepG2 cells with insulin resistance and oxidative damage. Data are expressed as mean ± SD (n = 3).

| Group | Concentration (µg/mL) | GOD (U/10^4^ cell) |
| --- | --- | --- |
| Control | - | 0.000 ± 0.009 |
| Model | - | 0.136 ± 0.007 |
| PSPC | 100 | 0.138 ± 0.008 |
|  | 40 | 0.136 ± 0.007 |
|  | 20 | 0.137 ± 0.008 |
| PSPW | 100 | 0.116 ± 0.016 |
|  | 40 | 0.119 ± 0.008 |
|  | 20 | 0.119 ± 0.003 |

**Table** **S3** Effects of PSPC and PSPW on body weight in T2DM rats. Data are expressed as mean ± SD (n = 9).

| Group | Body weight (g) | | | | | | |
| --- | --- | --- | --- | --- | --- | --- | --- |
|  | 0 d | 5 d | 10 d | 15 d | 20 d | 25 d | 30 d |
| Control | 253.67 ± 25.11 | 296.11 ± 39.61 | 317.44 ± 48.17 | 328.11 ± 46.75 | 346.33 ± 57.79 | 356.00 ± 53.54 | 369.71 ± 37.77 |
| Model | 248.20 ± 19.67 | 241.40 ± 27.14 | 245.20 ± 29.83 | 233.43 ± 26.59 | 218.17 ± 26.10 | 217.60 ± 30.08 | 202.67 ± 18.56 |
| Positive | 247.57 ± 13.40 | 232.89 ± 27.08 | 243.38 ± 20.37 | 249.11 ± 34.61 | 250.22 ± 29.98 | 265.33 ± 32.68 | 281.67 ± 23.49 |
| PSPW_H | 245.13 ± 21.27 | 238.71 ± 17.03 | 244.56 ± 32.05 | 235.20 ± 16.99 | 251.00 ± 19.83 | 260.50 ± 40.26 | 279.67 ± 27.01 |
| PSPW_L | 253.60 ± 13.78 | 245.89 ± 20.94 | 252.44 ± 22.95 | 235.50 ± 14.22 | 237.33 ± 18.96 | 256.89 ± 28.96 | 266.40 ± 20.82 |
| PSPC_H | 241.33 ± 14.73 | 236.29 ± 28.42 | 235.78 ± 39.45 | 230.00 ± 31.23 | 238.89 ± 41.46 | 255.11 ± 36.38 | 266.33 ± 27.79 |
| PSPC_L | 251.14 ± 17.55 | 244.43 ± 19.91 | 253.25 ± 23.66 | 250.38 ± 26.72 | 256.50 ± 24.66 | 268.33 ± 24.15 | 258.57 ± 20.07 |

**Table** **S4** Effects of PSPC and PSPW on FBG in T2DM rats. Data are expressed as mean ± SD (n = 9).

| Group | FBG (mmol/L) | | | |
| --- | --- | --- | --- | --- |
|  | 1 week | 2 weeks | 3 weeks | 4 weeks |
| Control | 9.7 ± 0.5 | 6.6 ± 0.8 | 6.1 ± 0.7 | 7.0 ± 1.4 |
| Model | 23.2 ± 0.9^**^ | 23.6 ± 4.9^**^ | 24.6 ± 4.4^**^ | 29.0 ± 2.7** |
| Positive | 23.6 ± 6.2 | 27.6 ± 3.5^#^ | 24.5 ± 0.7 | 18.9 ± 3.4^##^ |
| PSPW_H | 21.7 ± 2.6^&^ | 25.3 ± 4.7^#&^ | 23.9 ± 5.3^&^ | 20.0 ± 3.1^##&&^ |
| PSPW_L | 22.7 ± 3.7 | 24.6 ± 2.1 | 25.8 ± 1.7 | 21.4 ± 2.9^#&^ |
| PSPC_H | 26.9 ± 4.6^&^ | 22.4 ± 2.2 | 25.6 ± 2.1 | 24.3 ± 4.8^#^ |
| PSPC_L | 22.1 ± 3.4 | 24.6 ± 5.0 | 26.5 ± 3.6^#^ | 25.9 ± 3.9^#^ |

**P* < 0.05 and ** *P* < 0.01, significantly different from control; *^#^P* < 0.05 and *^##^* *P* < 0.01, significantly different from model; *^&^P* < 0.05 and *^&&^P* < 0.01, significantly different from PSPC.

**Table** **S5** Effects of PSPC and PSPW liver and kidney index in T2DM rats. Data are expressed as mean ± SD (n = 9).

| Group | Liver index | Kidney index |
| --- | --- | --- |
| Control | 2.52 ± 0.23 | 0.76 ± 0.02 |
| Model | 3.76 ± 0.08^**^ | 1.15 ± 0.09^**^ |
| Positive | 3.38 ± 0.09^#^ | 0.94 ± 0.06^#^ |
| PSPW_H | 3.41 ± 0.19^##^ | 1.00 ± 0.05^#^ |
| PSPW_L | 3.53± 0.22^#^ | 1.02 ± 0.15^#^ |
| PSPC_H | 3.53 ± 0.14^##^ | 1.10 ± 0.09^##^ |
| PSPC_L | 3.64 ± 0.27 | 1.12 ± 0.12^#^ |

**P* < 0.05 and ***P* < 0.01, significantly different from control; *^#^P* < 0.05 and *^##^P* < 0.01, significantly different from model.

**Table** **S6** Effects of PSPC and PSPW on TG, TC, GSP, NEFA, and INS in T2DM rats. Data are expressed as mean ± SD (n = 9).

| Group | TG (mmol/L) | TC (mmol/L) | GSP (mmol/L) | NEFA (mmol/L) | INS (ng/mL) |
| --- | --- | --- | --- | --- | --- |
| Control | 0.393 ± 0.078 | 1.245 ± 0.069 | 0.853 ± 0.109 | 0.252 ± 0.069 | 2.42 ± 0.50 |
| Model | 1.092 ± 0.230^**^ | 1.804 ± 0.267^**^ | 1.680 ± 0.253^**^ | 0.858 ± 0.185^**^ | 0.62 ± 0.17^**^ |
| Positive | 0.408 ± 0.086^##^ | 1.360 ± 0.157^#^ | 1.160 ± 0.123^##^ | 0.358 ± 0.098^##^ | 1.62 ± 0.50^##^ |
| PSPW_H | 0.440 ± 0.021^##&^ | 1.350 ± 0.110^##^ | 1.140 ± 0.080^##&^ | 0.320 ± 0.160^##&^ | 1.31 ± 0.17^##&&^ |
| PSPW_L | 0.460 ± 0.120^##&^ | 1.460 ± 0.160^#^ | 1.260 ± 0.130^#&^ | 0.390 ± 0.110^##&^ | 1.05 ± 0.55^##&^ |
| PSPC_H | 0.532 ±0.081^##^ | 1.489 ± 0.098^#^ | 1.318 ± 0.093^##^ | 0.570 ± 0.104^##^ | 0.85 ± 0.40^#^ |
| PSPC_L | 0.596 ± 0.057^##^ | 1.567 ± 0.089^#^ | 1.460 ± 0.216^#^ | 0.683 ± 0.107^#^ | 0.79 ± 0.28^#^ |

**P* < 0.05 and ** *P* < 0.01, significantly different from control; *^#^P* < 0.05 and *^##^* *P* < 0.01, significantly different from model; *^&^P* < 0.05 and *^&&^P* < 0.01, significantly different from PSPC.

**Table** **S7** Effects of PSPC and PSPW on SOD, CAT, GSH, and MDA in T2DM rats. Data are expressed as mean ± SD (n = 9).

| Group | SOD (U/mgprot) | CAT (U/mgprot) | GSH (µmol/mgprot) | MDA (mmol/ mgprot) |
| --- | --- | --- | --- | --- |
| Control | 100.00 ± 16.80 | 100.00 ± 11.01 | 100.00 ± 11.01 | 100.00 ± 18.00 |
| Model | 77.88 ± 9.07 | 79.65 ± 3.77 | 71.08 ± 10.16 | 136.82 ± 18.67 |
| Positive | 93.38 ± 9.84 | 92.88 ± 15.56 | 97.19 ± 11.25 | 119.38 ± 17.24 |
| PSPW_H | 91.73 ± 14.75 | 93.61 ± 11.41 | 90.64 ± 12.44 | 104.31 ± 8.42 |
| PSPW_L | 86.37 ± 12.41 | 82.31 ± 19.00 | 84.48 ± 13.99 | 110.39 ± 10.72 |
| PSPC_H | 82.70 ± 8.17 | 91.34 ± 11.27 | 82.46 ± 8.17 | 113.34 ± 16.38 |
| PSPC_L | 80.57 ± 11.09 | 78.21 ± 12.20 | 75.57 ± 11.09 | 119.57 ± 23.67 |

^b^*P* < 0.01, significantly different from control; ^c^*P* < 0.05 and ^d^*P* < 0.01, significantly different from model; ^e^*P* < 0.05 and ^f^*P* < 0.01, significantly different from PSPC.

**Table** **S8** Relative mRNA expression levels of PI3K, AKT, INSR, and GLUT-2 in rats, as determined by RT-qPCR. Data are expressed as mean ± SD (n = 3).

| Group | PI3K | AKT | INSR | GLUT-2 |
| --- | --- | --- | --- | --- |
| Control | 100.00 ± 4.00 | 100.00 ± 4.00 | 100.00 ± 4.00 | 100.00 ± 4.00 |
| Model | 69.50 ± 8.17 | 61.76 ± 11.02 | 54.22 ± 10.11 | 54.02 ± 9.65 |
| Positive | 89.29 ± 10.83 | 88.55 ± 10.44 | 80.22 ± 10.33 | 80.66 ± 10.27 |
| PSPW_H | 85.54 ± 8.20 | 89.36 ± 10.87 | 84.00 ± 10.20 | 75.22 ± 14.09 |
| PSPW_L | 79.65 ± 11.89 | 82.85 ± 11.54 | 80.21 ± 11.66 | 70.55 ± 10.65 |
| PSPC_H | 78.32 ± 7.16 | 76.35 ± 6.65 | 78.32 ± 14.16 | 68.74 ± 5.34 |
| PSPC_L | 72.57 ± 13.67 | 70.53 ± 11.98 | 69.58 ± 11.67 | 62.43 ± 13.42 |

^b^*P* < 0.01, significantly different from control; ^c^*P* < 0.05 and ^cc^*P* < 0.01, significantly different from model; ^d^*P* < 0.05 and ^e^*P* < 0.01, significantly different from PSPC.

**Table** **S9** Protein expression levels of p-IRS1, PI3K, p-AKT, mTOR, SREBP, and GLUT-2 in rats. Data are expressed as mean ± SD (n = 3).

| Group | p-IRS1/IRS1 | PI3K/β-actin | p-AKT/AKT | mTOR/β-actin | SREBP/β-actin | GLUT-2/β-actin |
| --- | --- | --- | --- | --- | --- | --- |
| Control | 18.155 ± 1.278 | 0.679 ± 0.062 | 1.539 ± 0.325 | 1.219 ± 0.188 | 1.806 ± 0.452 | 1.614 ± 0.258 |
| Model | 12.859 ± 1.520^**^ | 0.370 ± 0.129^*^ | 0.874 ± 0.168^*^ | 1.785 ± 0.132^*^ | 3.528 ± 0.782^*^ | 1.006 ± 0.225^*^ |
| Positive | 10.490 ± 4.537 | 1.294 ± 0.196^##^ | 1.639 ± 0.334^#^ | 1.531 ± 0.457 | 2.580 ± 0.559 | 1.779 ± 0.373^*^ |
| PSPW_H | 10.748 ± 2.004^#^ | 0.758 ± 0.379 | 1.328 ± 0.206^#^ | 1.344 ± 0.386 | 1.720 ± 0.560^#^ | 1.549 ± 0.240^*^ |
| PSPW_L | 27.458 ± 3.545^##^ | 0.818 ± 0.180^#^ | 1.532 ± 0.317^#^ | 1.187 ± 0.322^#^ | 1.720 ± 0.737^#^ | 1.253 ± 0.104 |
| PSPC_H | 19.953 ± 2.825^#^ | 0.709 ± 0.142^#^ | 1.323 ± 0.179^#^ | 1.416 ± 0.484 | 2.648 ± 0.914 | 1.510 ± 0.644 |
| PSPC_L | 22.878 ± 5.255^#^ | 0.977 ± 0.186^##^ | 1.368 ± 0.250^#^ | 1.845 ± 0.684 | 1.737 ± 0.795^#^ | 1.698 ± 0.329^*^ |

**P* < 0.05 and ***P* < 0.01, significantly different from control; ^#^*P* < 0.05 and ^##^*P* < 0.01, significantly different from model.
